# Pea protein-inulin conjugate prepared by atmospheric pressure plasma jet combined with glycosylation: structure and emulsifying properties

**DOI:** 10.3389/fnut.2024.1416753

**Published:** 2024-05-17

**Authors:** Hongfang Ji, Qingqing Wang, Xuefei Wang, Lingwen Zhang, Ping Yang

**Affiliations:** School of Food Science, Henan Institute of Science and Technology, Xinxiang, China

**Keywords:** atmospheric pressure plasma jet, discharge power, pea protein-inulin glycosylation conjugate, solubility, emulsifying ability, structure

## Abstract

Pea protein is one of plant proteins with high nutritional value, but its lower solubility and poor emulsifying properties limit its application in food industry. Based on wet-heating glycosylation of pea protein and inulin, effects of discharge power of atmospheric pressure plasma jet (APPJ) on structure, solubility, and emulsifying ability of pea protein-inulin glycosylation conjugate were explored. Results indicated that the APPJ discharge power did not affect the primary structure of pea protein. However, changes in secondary and spatial structure of pea protein were observed. When APPJ discharge power was 600 W, the solubility of glycosylation conjugate was 75.0% and the emulsifying stability index was 98.9 min, which increased by 14.85 and 21.95% than that of only glycosylation sample, respectively. These findings could provide technical support for APPJ treatment combination with glycosylation to enhance the physicochemical properties of plant-based proteins.

## 1 Introduction

Pea, the second soybean crop, is grown in more than 60 countries worldwide, and its annual production in China is about 1.5 million tons. Pea protein (PP), accounting for 23–25% of pea seeds (dry weight, D.W.), is rich in lysine and lack of sulfur-containing amino acids, and its amino acid composition is near to the standard model recommended by FAO/WHO (1). PP is easy to obtain with low cost, and has lower allergenicity than other legume proteins (2). PP also has physiological functions such as antioxidant activity, lowering blood pressure, and regulating intestinal flora (3). Therefore, PP is widely accepted. However, the solubility and emulsifying properties of PP were poor (4, 5). The lower solubility of PP is mainly due to the higher content of globulin, which accounts for about seven-tenth of the total protein. In addition, the alkali dissolution and acid precipitation method for separating pea protein from pea flour reduces its solubility (6, 7). In order to solve the above application limitations of PP and broaden its usage in the modern food processing, it is necessary to adopt the reasonable methods to modify PP (8, 9). Maillard reaction has been reported to effectively improve functional properties of food proteins (10). It was a safe and efficient method that occurred naturally and spontaneously under the controlled conditions such as reaction temperature, reaction time, and pH value without adding extraneous chemicals (10, 11). In spite of that, the traditional Maillard reaction (wet-heating conditions, and dry-heating conditions) has some drawbacks such as long heating time, and easy to cause protein denaturation and aggregation. The exploration of emerging technology-assisted Maillard reaction has attracted extensive attention of researchers (12–14).

Inulin is a kind of soluble dietary fiber, which is a chain polysaccharide composed of D-fructose residues linked by β-(2-1) glycosidic bonds. Its degree of polymerization is generally 2–60 (15). Inulin has the properties of stable chemical properties, strong water absorption and not easy to be decomposed by human digestive enzymes. Additionally, it has the functions of reducing blood lipids, lowering blood glucose, and promoting mineral absorption, and so on. In 2009, inulin was formally approved as a new resource food by Ministry of Health P. R. China. As the dietary fiber with the highest recognition, the largest market share and the widest application field in the world, inulin is widely used in baked goods, dairy products, candies, and beverages (16). In recent years, protein modification by Maillard reaction between inulin and protein has attracted much attention (17, 18). Whey protein isolate-inulin glycosylation conjugates were prepared by glycosylation under wet-heating condition, and its emulsifying activity and stability were significantly enhanced (17). Compared with the untreated walnut protein, the solubility and emulsifying index of the inulin-walnut protein glycosylation conjugate rose by 47 percent and 17 percent, respectively (18).

Cold plasma is usually called the fourth state of matter, which could be generated by different carrier gas systems (air, nitrogen, argon, etc.) at atmospheric or low pressure through radio frequency discharge, microwave discharges, atmospheric pressure jet discharge (APPJ), glow discharge, resistive barrier discharge, dielectric barrier discharge, and other different ways of discharge. Cold plasma is an electrically neutral ionized gas, including electrons, ions, and neutral particles in basic and excited states, and could trigger various chemical reactions to modifying the functional properties of food (19). DBD discharge cold plasma technology was employed to modify peanut protein, and it was reported that the content of both α-Helix and β-sheet decreased after treatment, and its ordered structure was destroyed (20). Compared with the untreated group, the solubility and water holding capacity of peanut protein isolate increased by 24.8 and 79.6%, respectively (21). Being treated by dielectric barrier discharge cold plasma under 12 kV of voltage, 30 W of power for 10 min, the emulsifying activity and stability of beef myofibrillar protein reached the maximum value (22). After being treated with non-thermal pin to plate cold plasma at atmospheric pressure, the percentage of soluble protein of pea protein isolate increased by 66.94 percent in the optimum treatment group compared with that of the untreated pea protein isolate group, and emulsifying activity and stability significantly increased, too (23). Previous literature has shown that cold plasma has great potential in modifying the physicochemical properties of protein. However, there were few published reports about the structural and functional properties changes of protein glycosylation conjugates treated by cold plasma.

The present study was, on basis of our previous assay, the influence of APPJ treatment with different discharge power on the solubility, emulsifying properties, secondary structure, and spatial structure of pea protein-inulin glycosylation conjugate were investigated in this paper. This study could provide certain theoretical basis for improving solubility and emulsifying ability of pea protein glycosylation conjugates by non-thermal processing technology, and broadening the application field of plant-based protein in food processing.

## 2 Materials and methods

### 2.1 Materials and chemicals

Pea protein (with protein content of 81.5%, ash content of 5.7%, fat content of 6.7%, carbohydrate content of 6.1%, based on dry weight) was from Shandong Jindu Talin Foods Co., Ltd. (Zhaoyuan, China); Inulin (DP value: 2–60, with inulin content of 92%) was the product of Baiyin Xirui Biological Engineering Co., Ltd. (Baiyin, China); Soybean oil was purchased from Longda Foodstuff Group Co., Ltd. (Laiyang, China). Other chemicals were analytical grade.

### 2.2 Preparation of pea protein-inulin glycosylation conjugate

**Figure 1:**
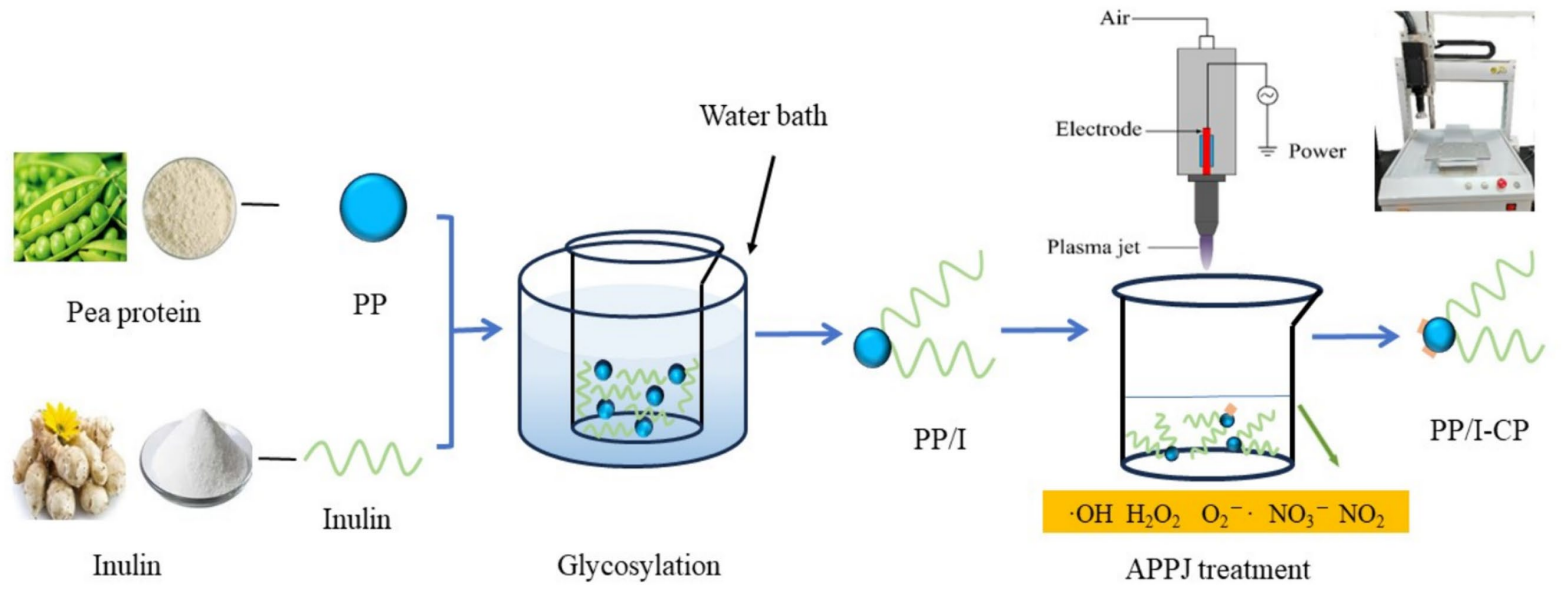
The process of APPJ treatment combination with glycosylation. PP refers to pea protein, PP/I refers to pea protein-inulin glycosylation conjugates, and PP/I-CP refers to pea protein-inulin glycosylation conjugates treated by APPJ.

Tweleve grams pea protein and 7.2 g inulin were suspended in 300 mL distilled water, followed by magnetic stirring at ambient temperature (20°C) for 30 min. And then, the pH value of the obtained mixture solution was adjusted to 9.0 by using sodium hydroxide solution (0.1 mol/L) or HCl solution (0.1 mol/L). Subsequently, the mixture was hydrated for 24 h at 4.0 ± 1.0°C, the suspension was heated for 90 min at 82.5°C with water bath. After being immediately cooled to about 20°C with ice-water bath, pea protein-inulin glycosylation conjugates (PP/I) suspension was obtained. PP/I suspension was treated in APPJ device (Henan Xiantu Zhineng Co., Ltd., Zhengzhou, China) for 270 s with different discharge power. PP/I and APPJ treated pea protein-inulin glycosylation conjugates (PP/I-CP) were freeze-dried. APPJ treated pea protein-inulin glycosylation conjugates was named PP/I-CP5, PP/I-CP6, PP/I-CP7, and PP/I-CP8 with discharge power of 500, 600, 700, and 800 W, respectively. Pea protein without any treatment was termed as PP. The process of APPJ treatment combination with glycosylation was displayed in Figure 1.

### 2.3 Solubility measurement

The solubility of protein samples was measured using the published method of Wang et al. (24) with minor modifications. One mg/mL of sample solution (PP, PP/I, and PP/I-CP) was prepared. After being stirred for 2 h at 20°C, the solution was centrifuged (3-30KS, Sigma, German) for 15 min at 8000 r/min. One millilitre of the supernatant was added to 4 mL biuret reagent. After vortex mixing and reaction for 30 min, the absorbance of pea protein sample solution was determined at 540 nm by UV2400 ultraviolet spectrophotometer (Shanghai Sunny Hengping Instrument, China). The solubility was calculated.

### 2.4 Emulsifying activity and emulsifying stability

The emulsifying activity index (EAI) and emulsifying stability index (ESI) of PP, PP/I, and PP/I-CP were detected according to the published method of Tang et al. (25) with slight modification. Briefly, 28 mL of 15 mg/mL protein sample solution was mixed with 7 mL of soybean oil, and then sheared by Ultra-turrax T-25 high-speed blender (IKA, Germany) for 20 s at 6000 r/min. Thirty microlitres of the protein sample emulsion was added to 15 mL of 1 mg/mL SDS solution. The absorbance was determined at 500 nm by using a UV2400 ultraviolet spectrophotometer. After the emulsion was stood for 5 min at ambient temperature, the same treatment was performed. EAI and ESI of PP, PP/I, and PP/I-CP was calculated according to the reported formulae (25).

### 2.5 Zeta-potential

The ζ-potential of PP, PP/I, and PP/I-CP was evaluated according to the literature reported by Xia et al. (26). Firstly, the protein samples were dispersed in phosphate buffer to achieve a final concentration of 0.1 mg/mL. And then, 1 mL of protein sample was analyzed using Zetasizer-Nano-ZS analyzer (Malvern, United Kingdom). The measurement temperature was 25°C.

### 2.6 SDS-PAGE

The changes in protein patterns of PP, PP/I, and PP/I-CP were evaluated by using SDS-PAGE according to the procedure of Zha et al. (7) with minor modification. In brief, the separating gel of 12% and stacking gel of 5% (Willget, Shanghai, China) was applied. Sixty microlitres of 1 mg/mL protein sample was mixed with 60 μL of loading buffer. And then the mixture was heated with water bath for 10 min at 95°C. The loading volume was 10 μL. The DYCZ-24DN protein electrophoresis cell (Beijing Liuyi Biotechnology Co., Ltd., China) was used. During electrophoresis, the voltage was kept constantly, the voltage of stacking gel was 110 V, and that of separating gel was 80 V. The gel was stained firstly and then destained after electrophoresis. The fixing solution was prepared with glacial acetic acid, CH_3_OH, and redistilled water with the volume ratio of 1:5:5. The staining solution was obtained by dissolving 250 mg of Coomassie brilliant blue (R-250) into 500 mL of fixing solution. The destained solution was prepared with glacial acetic acid, CH_3_OH, and redistilled water with the volume ratio of 2:3:35. The image was obtained by using Bio-rad GelDoc XR+ gel imaging system (Bio-rad, United States).

### 2.7 Infrared spectrum

The FTIR spectra were depicted by using a Bruker Tensor 27 spectrometer according to the published method of Li et al. (27). One milligram protein sample and 100 mg KBr was thoroughly mixed, and compressed into tablets under infrared lamp. The scanning wavelength range was from 4,000 to 400 cm^−1^, and the resolution was 4 cm^−1^. The FTIR spectra were obtained and the structure changes of protein samples were analyzed.

### 2.8 Intrinsic fluorescence

The intrinsic emission fluorescence spectra were determined by using Cary Eclipse fluorescence spectrophotometer (Varian, United States) according to the previously published method described by Jiang et al. (28) with slight modification. Solutions (2.5 mg/mL, in 0.2 mol/L phosphate buffer saline, pH 7.0) were excited at 280 nm. Wavelength of scanning was set in the range from 300 to 400 nm. The slit for excitation and emission was 10 nm. The phosphate buffer with pH value of 7.0 was used as the blank.

### 2.9 Surface hydrophobicity

Surface hydrophobicity of PP, PP/I, and PP/I-CP was measured according to reference of Chelh et al. (29). Five mg/mL sample solution was prepared with 0.01 mol/L phosphate buffer solution. Then, 1 mL of the upper sample solution was mixed with 200 μL of 1 mg/mL bromophenol blue. After the mixture was centrifuged at 6000 r/min for 20 min, 300 μL of supernatant was diluted to 10-fold with phosphate buffer solution. Then the absorbance of the mixture was measured by a UV2400 ultraviolet spectrophotometer (Shanghai Sunny Hengping Instrument, China) at 595 nm. The absorbance of PP, PP/I, and PP/I-CP sample and the control was obtained. For A_Control_, the 0.01 mol/L phosphate buffer solution was used instead of PP sample supernatant. Bromophenol blue binding capacity was calculated using the following formula.

### 2.10 Statistical analysis

All measurements were carried out in triplicate. Results were displayed as the mean ± standard deviation (SD). Significance analysis was based on the least significant difference (LSD) test by SPSS 25.0, and plotting was used by Origin 9.0 software. And the significance was set at *p* < 0.05.

## 3 Results and discussion

### 3.1 Effect of APPJ on solubility of pea protein-inulin glycosylation conjugates

**Figure 2:**
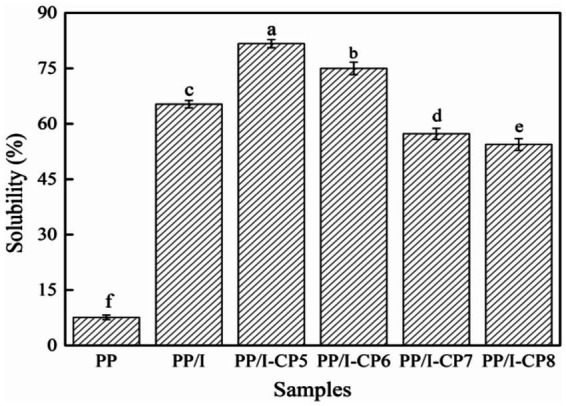
Solubility of pea protein-inulin glycosylation conjugates treated with APPJ at different discharge power. PP refers to pea protein, PP/I refers to pea protein-inulin glycosylation conjugates. PP/I-CP5, PP/I-CP6, PP/I-CP7, and PP/I-CP8 refers to pea protein-inulin glycosylation conjugates treated by APPJ with discharge power of 500, 600, 700, and 800 W, respectively. Different letters above the bar indicate the significant difference (*p* < 0.05).

Protein solubility plays an important role in the modern food industry. The effects of APPJ on solubility of PP, PP/I, and PP/I-CP were displayed in Figure 2. The solubility of pea protein-inulin glycosylation conjugates was 65.3%, which was significantly higher than that of the untreated pea protein (7.6%) (*p* < 0.05). Similar results were reported by Shen et al. (4) and Zha et al. (7). Shen et al. (4) reported that pea protein isolate-guar gum conjugates showed much higher solubility compared with the unmodified pea protein isolate. Zha et al. (7) found that, after glycosylation, the solubility of pea protein-gum arabic conjugates improved from 29.2 to 40.9%. The increase in solubility of protein might be due to the increased polarity of protein occurring the covalent linkage with the hydrophilic saccharides, therefore preventing the process of aggregation (30).

APPJ could obviously affect the solubility of PP/I (Figure 2). When the discharge power of APPJ treatment was 500 and 600 W, the solubility of PP/I was 81.7 and 75.0%, respectively, which increased by 25.11 and 14.85%, respectively, compared with that of PP/I without APPJ treatment. Under certain conditions, cold plasma treatment could introduce a great quantity of hydrophilic groups into the biological macromolecules, thus significantly increased the solubility (31). In addition, the surface structure of protein could be destroyed by cold plasma, its hydrophilic groups were exposed and the hydrophilicity increased, thereby accelerated the affinity of protein with water molecules and improved the solubility (32). When the discharge power of APPJ was more than 700 W, the solubility of PP/I decreased significantly, and was obviously lower than that of PP/I without APPJ treatment (*p* < 0.05). This might be attributed to the cross-linking between proteins caused by higher power discharge, and active sites of protein was reduced, which resulted in the decrease of protein solubility (12). In addition, the protein solubility was also affected by new oxygen-containing and nitrogen-containing groups on the surface of protein formed by the active species, which was produced by cold plasma (33).

### 3.2 Effect of APPJ on emulsifying property of pea protein-inulin glycosylation conjugates

**Figure 3:**
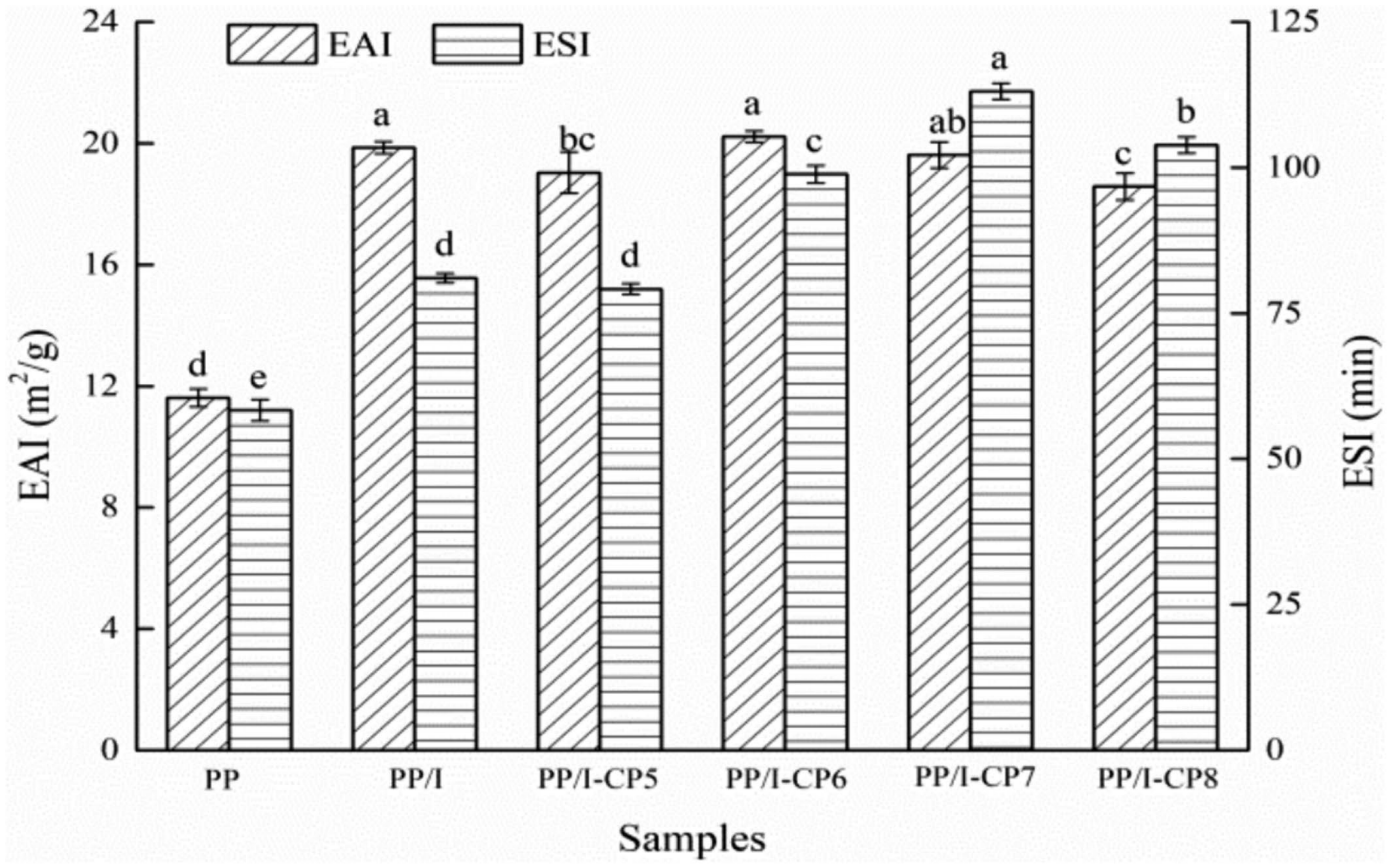
Emulsifying property of pea protein-inulin glycosylation conjugates treated with APPJ at different discharge power. PP refers to pea protein, PP/I refers to pea protein-inulin glycosylation conjugates. PP/I-CP5, PP/I-CP6, PP/I-CP7, and PP/I-CP8 refers to pea protein-inulin glycosylation conjugates treated by APPJ with discharge power of 500, 600, 700, and 800 W, respectively. Different letters above the bar indicate the significant difference (*p* < 0.05).

Emulsifying property is one of the measures to analyze the ability of protein forming and stabilizing emulsions (34). Compared with untreated pea protein, the emulsifying activity index of pea protein-inulin glycosylation conjugates obviously increased from 11.62 m^2^/g to 19.86 m^2^/g (*p* < 0.05), and the emulsifying stability index also increased remarkably (*p* < 0.05) (Figure 3). Similar results were also reported by Dong et al. (35) and Jiang et al. (36). After being glycosylated, the hydrophilic groups in polysaccharide chain were introduced and changed the spatial structure of pea protein. Protein glycosylation products could be used as amphiphilic surfactants, and rapidly diffused and adsorbed on the surface of oil droplets, which prevented the aggregation of oil droplets and improved the emulsifying properties of protein (37).

When APPJ discharge power was 500 W, both the emulsifying activity and emulsifying stability index of PP/I were lower than those of control (Figure 3), which indicated that certain number of hydrophilic groups were introduced into PP/I by cold plasma treatment, and thus made the emulsifying property of PP/I decrease. When discharge power rose to 600 W, the emulsifying activity index of PP/I reached the maximum value of 20.22 m^2^/g, and the emulsifying stability increased to 98.9 min, which indicated that the structure of PP/I became looser and more hydrophobic groups were exposed. However, when the discharge power exceeded 700 W, both the emulsifying activity and emulsifying stability index of PP/I decreased (Figure 3). This might be due to the crosslinking reaction between protein molecules caused by higher power discharge, and declining of the number of exposed hydrophobic groups. Sharifian et al. (22) reported that, after being modified by DBD cold plasma, both the emulsifying activity and emulsifying stability of myofibrillar protein were obviously promoted, while the emulsifying performance decreased when the modification time was longer than 20 min. Du et al. (38) found the similar result when they carried out the research about the influence of cold plasma at the reduced pressure on functional properties of soybean protein isolate.

### 3.3 Effect of APPJ on ζ-potential of pea protein-inulin glycosylation conjugates

Zeta potential is an index of total surface charge of protein and one of the important indexes to estimate the stability of protein dispersion system. The larger the absolute value of ζ-potential of protein was, the intenser the electrostatic repulsion was, and the more stable the protein dispersion system was (39).

**Figure 4:**
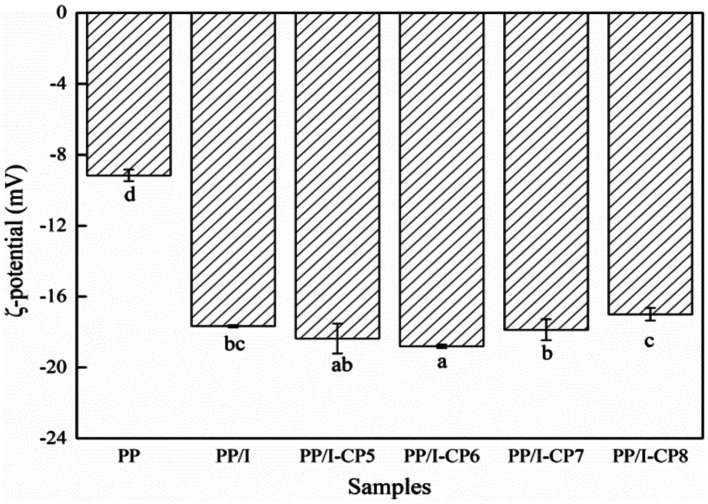
ζ-potential of pea protein-inulin glycosylation conjugates treated with APPJ at different discharge power. PP refers to pea protein, PP/I refers to pea protein-inulin glycosylation conjugates. PP/I-CP5, PP/I-CP6, PP/I-CP7, and PP/I-CP8 refers to pea protein-inulin glycosylation conjugates treated by APPJ with discharge power of 500, 600, 700, and 800 W, respectively. Different letters above the bar indicate the significant difference (*p* < 0.05).

The absolute value of ζ-potential of PP/I emulsion was remarkably higher than that of untreated pea protein (*p* < 0.05) (Figure 4). The same result was reported by Pirestani et al. (6). This indicated that covalent bonds between protein and polysaccharide could promote the extension of polypeptide chains and expose negative charges, and the repulsion between charges could make the protein solution system relatively stable (40).

Compared with PP/I, the absolute value of ζ-potential of both 500 W and 600 W APPJ treated pea protein glycosylation conjugates solution increased. When the discharge power was greater than 700 W, the absolute value of ζ-potential decreased (Figure 4). Chen et al. (41) found the similar result. This indicated that cold plasma treatment with higher discharge power could result in the cross-linking aggregation of protein, and destroy the balance between electrostatic repulsion and electrostatic attraction, and reduce the stability of protein emulsion.

### 3.4 SDS-PAGE profile of pea protein-inulin glycosylation conjugates

**Figure 5:**
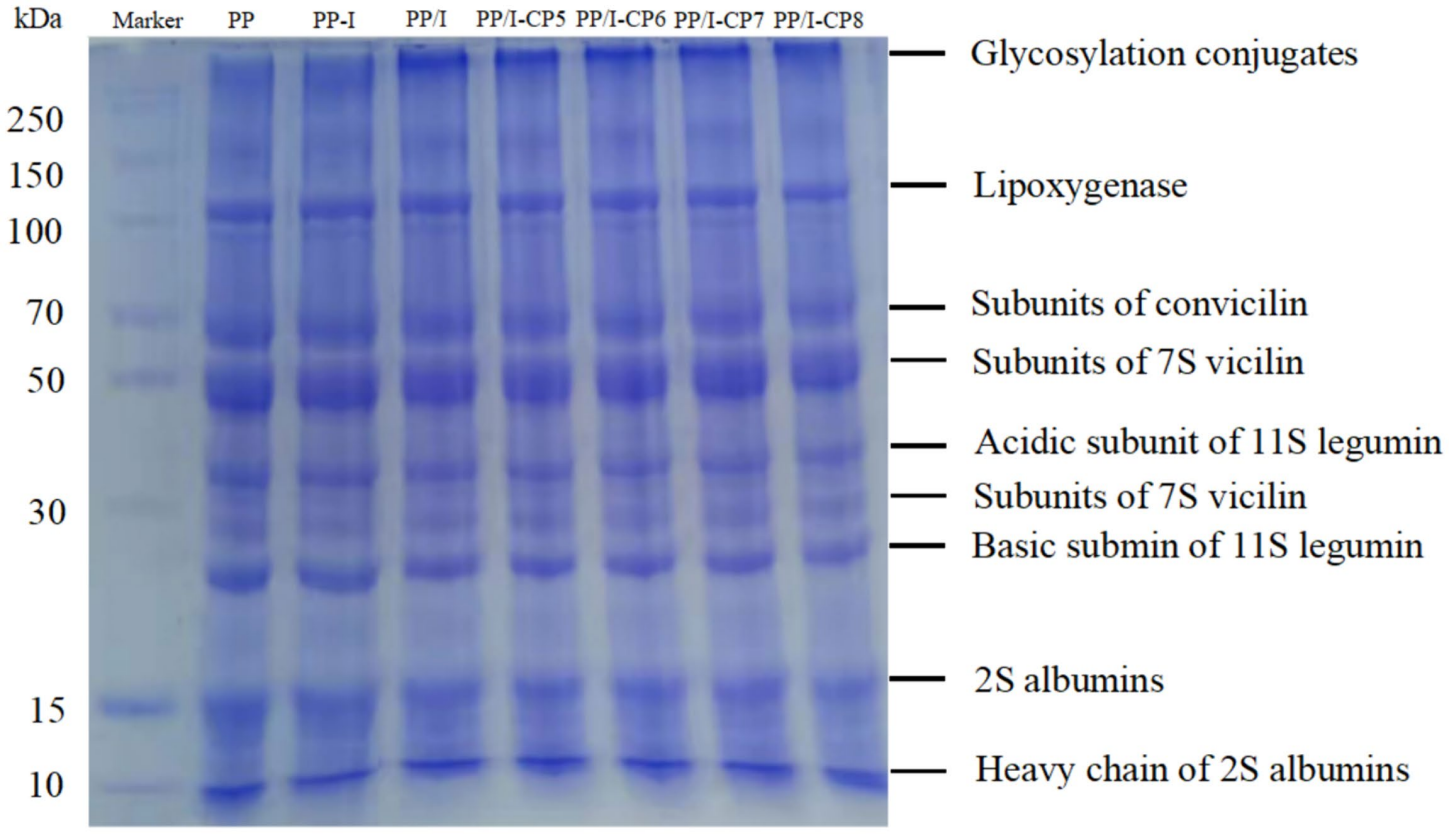
SDS-PAGE profile of pea protein-inulin glycosylation conjugates treated with APPJ at different discharge power. PP refers to pea protein, PP-I refers to pea protein-inulin simple mixture, PP/I refers to pea protein-inulin glycosylation conjugates. PP/I-CP5, PP/I-CP6, PP/I-CP7, and PP/I-CP8 refers to pea protein-inulin glycosylation conjugates treated by APPJ with discharge power of 500, 600, 700, and 800 W, respectively.

The molecular weight changes of PP, PP/I, and PP/I-CP were analyzed by using SDS-PAGE (Figure 5). The lane of untreated pea protein contained the following bands, lipoxygenase (~97 kDa), subunits of conviciline (~70 kDa), vicilin (~50, 33, and 28 kDa), acidic subunit of 11S legumin (~38 kDa), 2S albumin (~15 kDa). Similar result was reported by Zha et al. (7). No significant change was observed in the electrophoresis bands of inulin-pea protein simple mixture system (Figure 5, lane of PP-I), which revealed that simple physical blending could not influence the molecular weight distribution of PP.

Being glycosylated with inulin, a new band near the loading end of PP/I was detected (Figure 5, lane of PP/I), and it could not migrate into the separating gel, which was attributed to its larger molecular weight (>250 kDa). However, the band was not found in the untreated pea protein and PP-inulin simple mixture (Figure 5, lane of PP, and PP-I). Additionally, the band of PP/I at about 15 kDa (it was assigned to 2S albumin) increased compared to that of the unmodified pea protein. Maillard reaction between inulin and pea protein forming new substances with higher molecular weight could result in the above observations. Overall, the formation of inulin-pea protein glycosylation conjugates could be confirmed in the results of SDS-PAGE. Similar results of SDS-PAGE profile were in line with the reports by Pirestani et al. (6) and Jiang et al. (36).

Compared with only glycosylated pea protein-inulin conjugates, APPJ treatment did not cause significant changes in protein electrophoresis pattern. While, the intensity of polypeptides bands of near the loading end of the PP/I-CP dropped slightly as the change of APPJ discharge power (Figure 5). Similar changes were observed in the bands of acidic subunit of 11S legumin (~ 38 kDa) and basic submin of 11S legumin.

### 3.5 Fourier transform infrared spectroscopy analysis

**Figure 6:**
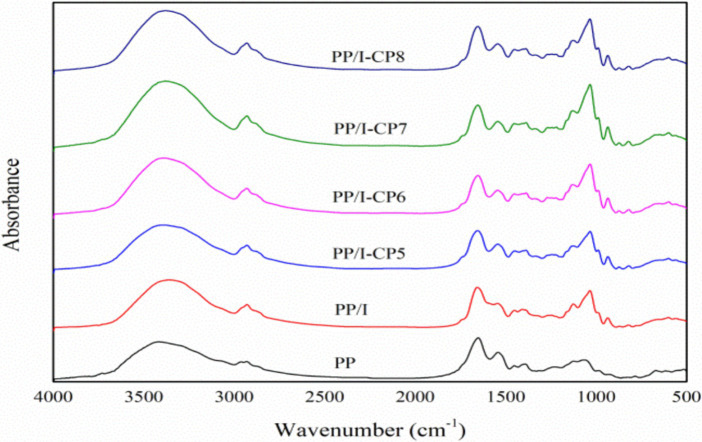
FTIR spectra of pea protein-inulin glycosylation conjugates treated with APPJ at different discharge power. PP refers to pea protein, PP/I refers to pea protein-inulin glycosylation conjugates. PP/I-CP5, PP/I-CP6, PP/I-CP7, and PP/I-CP8 refers to pea protein-inulin glycosylation conjugates treated by APPJ with discharge power of 500, 600, 700, and 800 W, respectively.

Fourier transform infrared spectroscopy (FTIR) is commonly employed to analyze protein structure. Bands at the regions of 1,260–1,000 cm^−1^ usually referred to C–O stretching (4). While the bands at 3,500–3,000 cm^−1^ were assigned to the –OH stretching vibration. The absorption in 1,260–1,000 cm^−1^ in PP/I sample was stronger than that in untreated pea protein (Figure 6), which demonstrated that covalent bonds were formed between pea protein and inulin. Additionally, it was observed that the peak intensity at the regions of 3,500–3,000 cm^−1^ in PP/I sample became intenser (Figure 6), which revealed that the hydrogen bonding and hydroxyl groups in pea protein-inulin conjugates surface increased obviously after Maillard reaction. Qu et al. (42) reported that the peak intensity at the regions of 3,500–3,000 cm^−1^ was significantly positive to the enhancement of protein solubility. This result was in accordance with the change of PP/I solubility, too (Figure 2).

**Figure 7:**
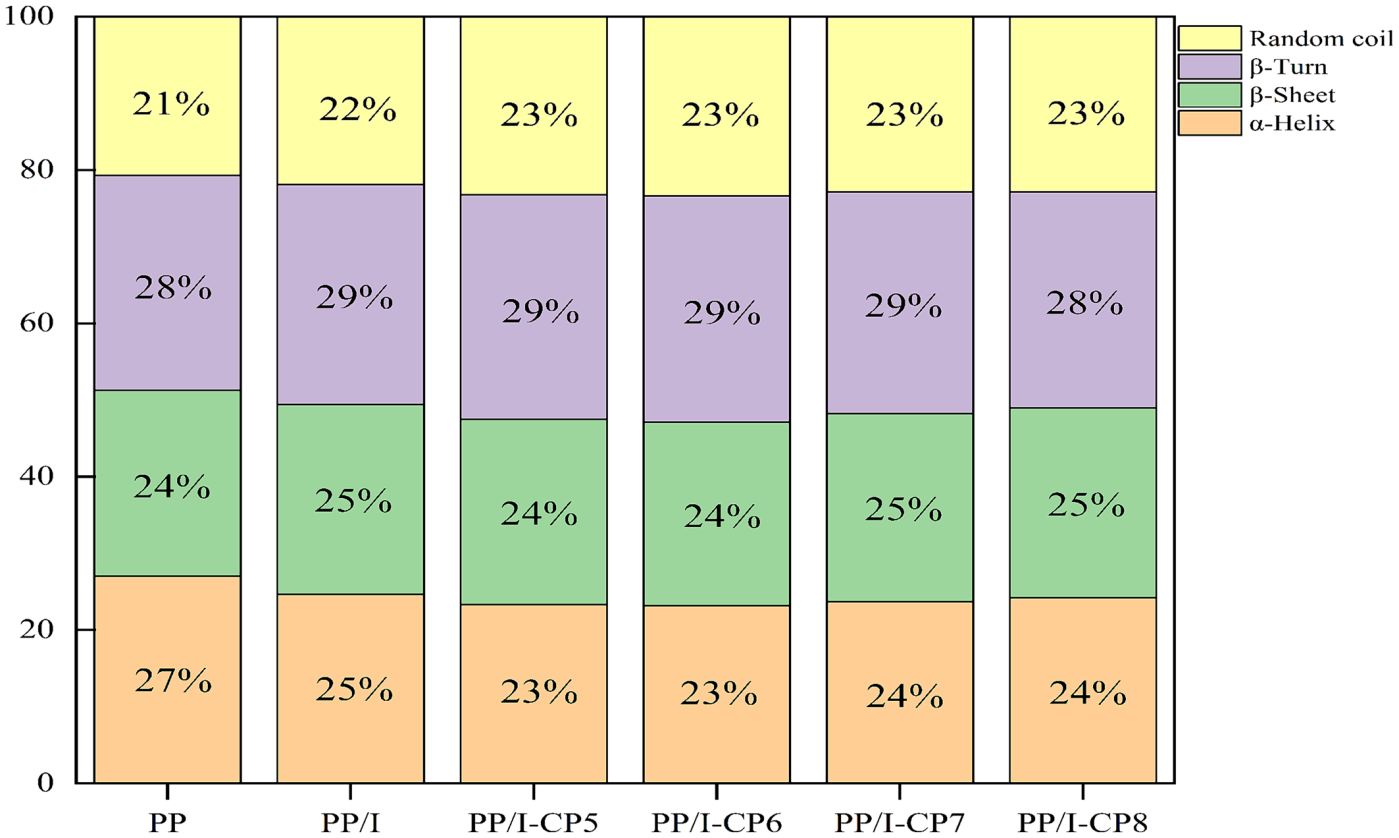
Secondary structure content of pea protein-inulin glycosylation conjugates treated with APPJ at different discharge power. PP refers to pea protein, PP/I refers to pea protein-inulin glycosylation conjugates. PP/I-CP5, PP/I-CP6, PP/I-CP7, and PP/I-CP8 refers to pea protein-inulin glycosylation conjugates treated by APPJ with discharge power of 500, 600, 700, and 800 W, respectively.

The amide I region absorption band of protein in the range of 1700–1,600 cm^−1^ could reflect the change of secondary structure. After being glycosylated, α-Helix structure of pea protein-inulin conjugates decreased, while random coil increased (Figure 7). Similar results were reported by Hou et al. (43). Meng et al. (40) also demonstrated that the interaction between whey protein isolates and D-Tagatose led to a reduction in α-Helix. The glycosylation reaction occurred between the amino group in α-Helix and the reducing carbonyl group in polysaccharides, thereby led to the decline in content of α-Helix.

When APPJ discharge power was in range from 500 to 600 W, α-Helix and β-sheet contents of pea protein-inulin glycosylated products decreased, while β-turn and random coil contents increased with increasing discharge power of APPJ. When the treatment power was over 700 W, α-Helix and β-sheet contents rose slightly, while β-turn decreased (Figure 7). β-turn content is directly proportional to protein hydration properties. The increase of β-turn content could improve protein hydration properties, this was consistent with the results of protein sample solubility (Figure 2). The higher the random coil content of glycosylated protein was, the better its emulsifying properties was (44), this was consistent with the results of emulsifying (Figure 3).

### 3.6 Analysis of fluorescence spectra

Tryptophan and tyrosine are the main sources of intrinsic fluorescence of proteins, both of them emit fluorescence at the wavelength of 280 nm. The maximum emission wavelength of tryptophan residues is about 340 nm. When the micro-environment surrounding chromophore group tended to be polar, the maximum emission wavelength would shift to be red. The quenching agent (protein or solvent itself) could reduce the fluorescence intensity when it quenched the fluorescence of chromophore groups. It can be seen that the intrinsic fluorescence spectra could reflect the degree of change of tertiary structure (45).

**Figure 8:**
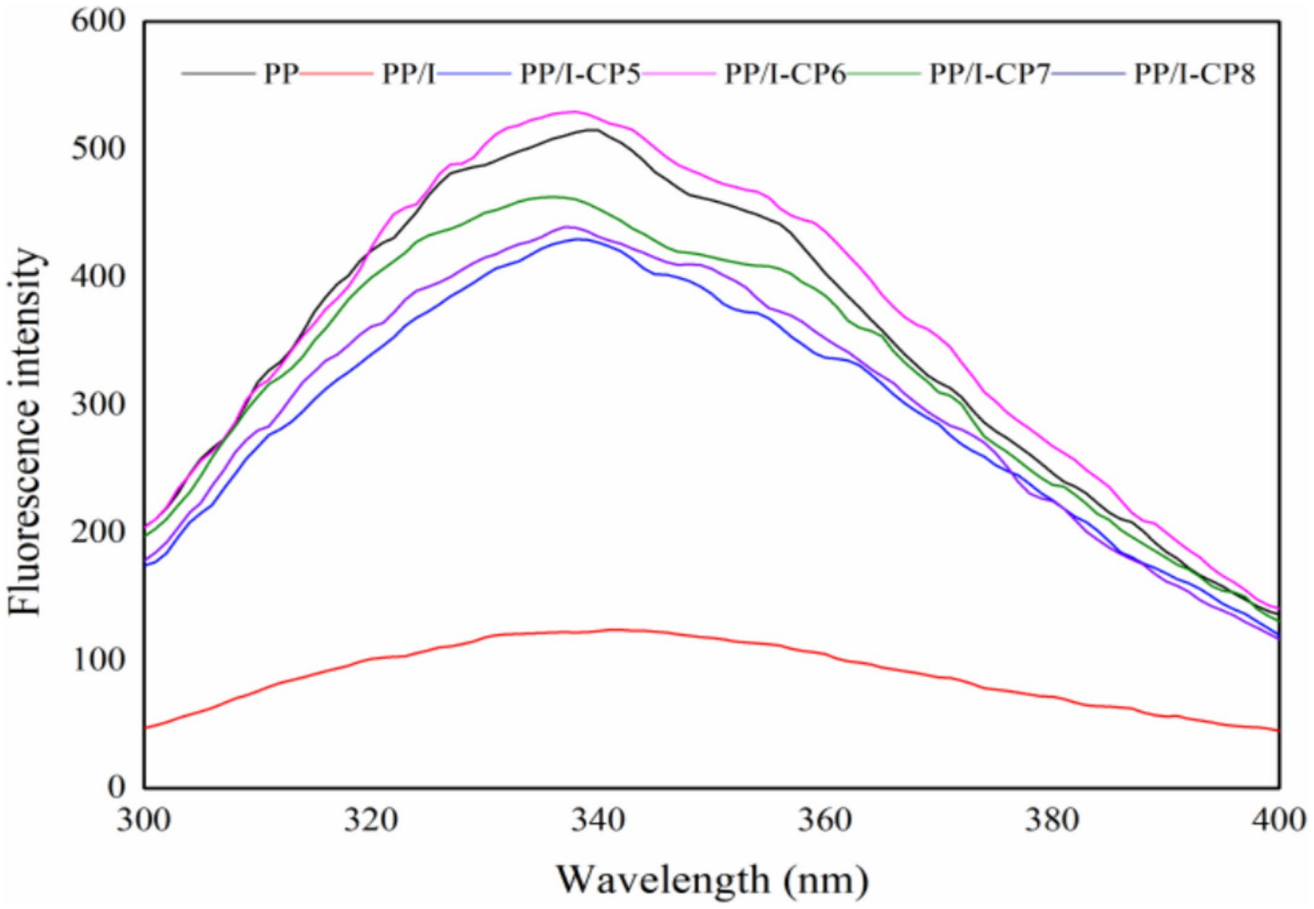
The intrinsic fluorescence spectra of pea protein-inulin glycosylation conjugates treated with APPJ at different discharge power. PP refers to pea protein, PP/I refers to pea protein-inulin glycosylation conjugates. PP/I-CP5, PP/I-CP6, PP/I-CP7, and PP/I-CP8 refers to pea protein-inulin glycosylation conjugates treated by APPJ with discharge power of 500, 600, 700, and 800 W, respectively.

The intrinsic fluorescence spectra of PP, PP/I, and PP/I-CP samples were plotted in Figure 8, which provided a clear distinction between the fluorescence intensity of the untreated pea protein and glycosylated pea protein. Similar results were reported by Spotti et al. (46) and Pirestanii et al. (47). This could be attributed to the shielding effect of polysaccharide chains in protein glycosylation products on tryptophan residues (48). Additionally, the maximum emission wavelength of untreated pea protein was at 339 nm, while that of PP/I was red-shifted to 342 nm. These results revealed that grafting reaction could change the conformation of protein, and loosen the tertiary structure of protein.

Compared with PP/I, the fluorescence intensity of APPJ treatment samples was obviously enhanced, and the fluorescence peak values of protein solution system were slightly blue-shifted (Figure 8). It was possible that cold plasma treatment could unfold the structure of protein, expose the hydrophobic groups buried in protein molecules to the surface, and enhance the number of the fluorescent chromophoric groups on the surface of PP/I-CP.

### 3.7 Effect of APPJ on surface hydrophobicity of pea protein-inulin glycosylation conjugates

The number of hydrophobic groups of proteins exposed in polar water environment was an important indicator of their surface hydrophobicity (49), and its changes could affect not only the structure, but also the functional properties of protein (50). Generally, when the structure of protein was loose, its surface hydrophobicity would be raised, and the aggregation and cross-linking reaction of protein would lead to the decline of the surface hydrophobicity. The binding content of bromophenol blue could be utilized to estimate the surface hydrophobicity of protein sample. Generally speaking, the larger the binding capacity of bromophenol blue of protein was, the higher its surface hydrophobicity was (51).

**Figure 9:**
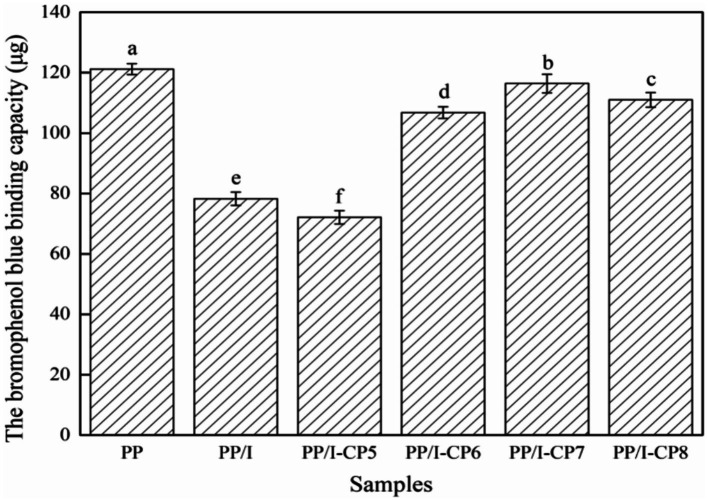
The surface hydrophobicity of pea protein-inulin glycosylation conjugates. Different letters above the bar indicate the significant difference (*p* < 0.05). PP refers to pea protein, PP/I refers to pea protein-inulin glycosylation conjugates. PP/I-CP5, PP/I-CP6, PP/I-CP7, and PP/I-CP8 refers to pea protein-inulin glycosylation conjugates treated by APPJ with discharge power of 500, 600, 700, and 800 W, respectively.

The bromophenol blue binding capacity of PP/I was 78.3 μg, which was significantly lower than that of the untreated pea protein (121.2 μg) (*p* < 0.05) (Figure 9). This is consistent with the results of Zhang et al. (52) and Ma et al. (53). Zhang et al. (52) found that the glycosylation could reduce the surface hydrophobicity of soybean protein-maltodextrin products. Ma et al. (53) revealed that glycosylation modification could inhibit the exposure of hydrophobic groups of soy protein isolate-pectin products. After glycosylation, the protein system produced a large number of hydrophilic groups, changed the balance between hydrophilicity and hydrophobicity, and shielded part of the surface hydrophobic groups, these resulted in the lower surface hydrophobicity.

With the enhancement of APPJ treatment discharge power, the surface hydrophobicity of pea protein rose gradually and then decreased. When APPJ discharge power was 700 W, the surface hydrophobicity reached the maximum value of 116.4 μg (Figure 9). With the increase of APPJ discharge power, the surface structure of the protein was destroyed by degrees, the protein structure became disordered and loose, a great number of hydrophobic groups were exposed, and the surface hydrophobicity of the protein was significantly improved. With the further increase of discharge power, the protein might be cross-linked, and the decline of the number of exposed hydrophobic groups resulted in the reduce of the bromophenol blue binding capacity. Similar phenomenon was found by Duan et al. (54), who observed that mild oxidation conditions could increase egg white proteins’ surface hydrophobicity due to the extension of the spatial structure, while excessive oxidation could reduce the surface hydrophobicity.

## 4 Conclusion

Using pea protein and inulin as raw materials, the effects of discharge powers of APPJ on the structure and emulsifying ability of pea protein-inulin glycosylation products were investigated. Subsequently, the changes in structure of pea protein-inulin glycosylation products were analyzed. APPJ treatment with certain discharge power could reduce the α-Helix content of pea protein-inulin glycosylation conjugates, and raise its random coil and β-turns content. Additionally, APPJ treatment could enhance the fluorescence intensity and affect the bromophenol blue binding capacity of PP/I. The solubility and emulsification stability of pea protein-inulin glycosylation products treated by APPJ with 600 W discharge power was significantly improved compared with those of the only wet-heat glycosylation product (*p* < 0.05). The future work on the influences of APPJ on the biological activity of pea protein-inulin glycosylation products will need to be carried out.

## Data availability statement

The original contributions presented in the study are included in the article/supplementary material, further inquiries can be directed to the corresponding author.

## Author contributions

HJ: Formal analysis, Funding acquisition, Investigation, Methodology, Project administration, Resources, Software, Supervision, Validation, Visualization, Writing – original draft, Writing – review & editing. QW: Formal analysis, Software, Validation, Visualization, Writing – original draft, Writing – review & editing. XW: Software, Writing – original draft, Writing – review & editing. LZ: Conceptualization, Funding acquisition, Methodology, Resources, Supervision, Writing – original draft, Writing – review & editing. PY: Data curation, Methodology, Validation, Writing – original draft, Writing – review & editing.
